# Sevoflurane requirement during elective ankle day surgery: the effects of etirocoxib premedication, a prospective randomised study

**DOI:** 10.1186/1749-799X-3-40

**Published:** 2008-09-11

**Authors:** Ibrahim Turan, Anette Hein, Eva Jacobson, Jan G Jakobsson

**Affiliations:** 1 Karolinska Institutet, Foot & Ankle Surgical Centre, Stockholm, Sweden; 2 Karolinska Institutet, Department of Anaesthesia, Danderyd Hospital, Stockholm, Sweden; 3 Karolinska Institutet, Institution for Physiology & Pharmacology, Department of Anaesthesia & Intensive Care, Foot & Ankle Surgical Centre, Stockholm, Sweden

## Abstract

**Background:**

Anti-inflammatory drugs, NSAIDs, have become an important part of the pain management in day surgery. The aim of the present study was to evaluate the effect of Coxib premedication on the intra-operative anaesthetic requirements in patients undergoing elective ankle surgery in general anaesthesia.

**Type of study:**

Prospective, randomized study of the intra-operative anaesthetic-sparing effects of etoricoxib premedication as compared to no NSAID preoperatively.

**Methods:**

The intra-operative requirement of sevoflurane was studied in forty-four ASA 1–2 patients undergoing elective ankle day surgical in balanced general anaesthesia.

Primary study endpoint was end-tidal sevoflurane concentration to maintain Cerebral State Index of 40 – 50 during surgery.

**Results:**

All anaesthesia and surgery was uneventful, no complications or adverse events were noticed. The mean end-tidal sevoflurane concentration intra-operatively was 1.25 (SD 0.2) and 0.91 (SD 0.2) for the pre and post-operative administered group of patients respectively (p < 0.0001). No other intra-operative differences could be noted. Emergence and recovery was rapid and no difference was noticed in time to discharge-eligible mean 52 minutes in both groups studied. In all 6 patients, 5 in the group receiving etoricoxib post-operatively, after surgery, and one in the pre-operative group required rescue analgesia before discharge from hospital. No difference was seen in pain or need for rescue analgesia, nausea or patients satisfaction during the first 24 postoperative hours.

**Conclusion:**

Coxib premedication before elective day surgery has an anaesthetic sparing potential.

## Background

Multi modal postoperative pain management has become standard of care especially in day case surgery [1]. Local anaesthesia applied prior to incision has been shown to have positive effects not only on the postoperative course but also to reduce the needs for main anaesthetic [2-4]. The effects of pre-treatment, premedication with NSAIDs as well as pre-incisional local anaesthesia have convincingly been shown to have a major impact on the postoperative pain course [5]. We found, in a previous study, pre-incisional local anaesthesia to have also clear intra-operative anaesthetic sparing effects [4]. The intra-operative effects, the effects on the main anaesthetic requirement, of preoperative administration of a Coxibs are, however, not well studied. Hypothetically administration of Coxibs preoperatively could have intra-operative effects by its prostaglandin inhibitory effect and thereby additive analgesic effects reducing the need for main anaesthetic.

The aim of the present study was to evaluate the effects of adding a Coxib pre-operatively on the need for main anaesthetic during balanced anaesthesia for elective day surgery.

## Methods

After informed consent 44 healthy American Society of Anesthesiology physiological status 1–2 patients; healthy patients aged 18 to 70 years scheduled for elective ankle surgery in general anaesthesia, as day cases, were eligible for inclusion. Patients with history of any previous reaction to NSAIDs or Coxibs, renal disease, not fully controlled cardiovascular disease or psychiatric disease requiring Litium therapy were excluded. Seventeen males and 27 females; mean age 45 (SD 14) years, mean weight 80 (SD 15) was included.

The study protocol, a prospective randomised study of the effects of pre-operative administration of a Coxib on the intra-operative anaesthetic requirements during elective day surgery was approved by ethical board at Karolinska Institut in Stockholm.

Primary study end-point: mean end-tidal sevoflurane concentration during surgery to maintain a Cerebral State index 40 – 50.

Secondary study variables;

• Need for rescue analgesics during recovery

• Need for rescue analgesia during the first 24 postoperative hour

• PONV

• Patients' satisfaction

The patients were randomised into two groups by computer prepared randomisation;

• Group A had etoricoxib 120 mg oral pre-surgery – preoperative group

• Group B had etoricoxib 120 mg oral right after end-of-surgery – postoperative group

All patients followed the routine pre and postoperative protocol of our institution.

They were asked to refrain from eating for 6 hours and drinking for 2 hour prior to surgery.

After establishment of an intravenous line all patients were given 8 mg betamethasone, and a sedation dose of 30 – 40 mg propofol; washing and dressing while sedated.

Two electrodes were applied on the forehead and one on the masteoid in accordance to the instructions for Cerebral State Monitor. The Cerebral State Index (CSI) derived from a Cerebral State Monitor (Danmeter A/S, Kildemosevej 13, DK-5000 Odense C, Denmark) was followed during the entire procedure.

All patients had induction of anaesthesia with a combination of 8–9 μg/kg alfentanil as a iv. injection and propofol sufficient to allow insertion of a laryngeal mask airway. All patients were breathing spontaneous; no muscle relaxant was given.

Right after insertion of the laryngeal mask sevoflurane in oxygen/air 1 L/min was introduced as main anaesthetic. During surgery a CSI of 40 – 50 was set as adequate depth of anaesthesia. The Sevoflurane was titrated aiming for a Cerebral State Index of 40 – 50 throughout surgery; the vaporizer setting was increased to 8% if signs of light anaesthesia and decreased stepwise by 2% if signs of excessive anaesthesia. Any other signs of inadequate or excessive anaesthesia by means of changes in heart rate, blood pressure or movements were also acknowledged. During wound close sevoflurane was discontinued and fresh gas flows were increased. All patients had local anaesthesia (bupivacaine 5 mg/ml 10 cc) in the wound area at end of surgery cc infiltrated in open surgery and injected in to the joint in arthroscopy patients in accordance to the routine of the department.

Cerebral State Index, end-tidal sevoflurane concentrations, heart rate, systolic blood pressure and respiratory rate were recorded every 3^rd ^minute during surgery. Mean end-tidal sevoflurane concentration during maintenance of anaesthesia was calculated as primary study variable.

Immediately after surgery all patients were moved to the recovery area, if fully awake and alert an Observer Assessment of Sedation Score of 5, possibly bypassing conventional recovery room stay. After arrival in recovery area all patients had an initial oral loading dose of 30 mg/kg paracetamol in accordance to the routines of the department. The postoperative group was together with the paracetamol given 120 mg etoricoxib orally; in the pre-operative group no further analgesics were given. Rescue analgesia, oxicodone orally, was provided in case patient graded pain on a 4 graded scale; no pain, mild pain, pain, severe pain. Patients were discharged when awake, and ambulant with minimal and acceptable levels of subjective pain (no/mild pain) in accordance with the standardised protocol of the institution.

At discharge all patients were provided with dextropropoxyphene 100 mg as oral rescue analgesic. All patients were also asked to fill in a postoperative questionnaire about pain, need for rescue analgesia, emesis, sleep disturbance and overall pain treatment satisfaction.

### Statistics

All values are given as mean and standard deviation. End-tidal concentration during maintenance and all other continuous data was compared between groups by ANOVA, differences in category data was analysed by Chi-Square test, and a p < 0.05 was considered statistical significant.

The number of patients, 22 in each group studied, was derived on a power calculation based on the assumption that in the control group, the group receiving the etoricoxib after surgery would have a mean end-tidal concentration of 1.2 with a standard deviation of 0.23 and that a reduction of 0.2 would be a clinically significant difference.

All statistics were made in StatView™ on a Macintosh computer system.

## Results

The groups were fully comparative; there was no significant difference in patient demographics Table 1. Time from etoricoxib premedication to start of anaesthesia was 25 (SD 4) minutes.

**Table 1:** Patient's demographics

	**Pre-treatment **(n = 22)	**Post-treatment **(n = 22)
***Sex (male/female)***	6/16	11/11
***Age (year.)***	47 ± 12	42 ± 15
***Weight (kg)***	80 ± 16	80 ± 15

***Surgery***		
*Peroneus ligament*	5	7
*Fibulo-talar ligament*	12	11
*Arthroscopy of the ankle*	5	4

All surgery and anaesthesia was uneventful and no complications or adverse effects were noticed. One patient in the etirocoxib group scheduled for arthroscopy had a conversion to open surgery in order to achieve effect resection of an exostosis but was still included in the analysis. Duration of procedures was also the same in both groups; mean duration of surgery (knife to skin to closed wound) was 21 (SD 7) minutes.

The mean end-tidal sevoflurane concentration to maintain a CSI of 46 (SD 3.9) showed a difference by 0.34% for the pre and post-operative administered group of patients respectively (p < 0.0001). No other differences were noticed intra-operatively; heart rate, blood pressure and respiratory rate were all well controlled during surgery and no other major signs of light or excessive anaesthesia were noticed. No additional analgesics were given during surgery.

Emergence was rapid and all patients were safely "fast-tracked", by-passing the regular recovery room, into the phase II recovery area. Recovery times, time to allowing drinking and to ambulate were the same in both groups. In all 6 patients required rescue analgesia during stay in the recovery area. Five patients in the group receiving etoricoxib post-operatively/after surgery and one patient in the pre-operative group required rescue analgesia before discharge. No patients complained about nausea during the stay in the recovery area. Eligible for discharge did not differ; all patients were discharged home safely within 80 minutes from reaching the phase II recovery area (Table 2).

**Table 2:** Intra and post operative observations

	**Pre-treatment **(n = 22)	**Post-treatment **(n = 22)
***Duration of surgery (min.)***	21 ± 6	21 ± 7
***Propofol (mg/kg)***	2.36 ± 0.45	2.41 ± 0.44
***Alfentanil μg/kg***	0.92 ± 0.09	0.96 ± 0.09
***CSI***	46 ± 5	46 ± 3
***Et Sevo mean (%)***	0.909 ± 0.17	1.246 ± 0.23 **
***Discharge (min.)***	52 ± 5	52 ± 9
***Analgesics before discharge (No. of Pat.)***	1/21	5/17

** p < 0.0001 ANOVA

Et Sevo; end tidal concentration of sevoflurane

Five patients were lost for follow-up, 3 in the pre-operative and 2 in the postoperative etoricoxib group of patients respectively. No significant difference was noticed in pain ratings or need for opioid rescue analgesia during the first 24 postoperative hours. Three patients in the pre-operative group and 5 in the postoperative group had at least one opioid rescue during the 24-hour follow-up period. Four and two patients in the pre and post treatment group respectively experienced PONV following discharge. No difference was seen in "patients' satisfaction" all thirty-nine patients followed graded postoperative pain management as satisfactory.

## Discussion

Our study is positive; we found a statistical significant difference between the pre- and post-operative administration of etoricoxib with regard to our primary study endpoint; mean end-tidal sevoflurane concentration needed to maintain a Cerebral State Index of 40–50 during elective minor day surgery.

The interpretation of our results should be done with caution, as the study design is complex. It should indeed be acknowledged that our patients received a multi-modal analgesic regime and balanced anaesthesia. All patients received both betamethasone and alfentanil at induction. The synergistic interaction between sevoflurane and opioids, reducing the "effective dose 50" (MAC) is well documented [6]. A clear sevoflurane sparing effect from small doses of fentanyl in day surgical anaesthesia has been shown both with and without monitoring of anaesthetic depth [7]. The main anaesthetic sparing effects of local anaesthesia is also well recognised [2,3]. We found in an earlier study, designed similar to the present, a clear effect from a peripheral local anaesthesia block on the sevoflurane requirement during minor orthopaedic day surgery, Hallux Valgus surgery [4]. Romunstad et al has also convincingly shown the analgesic properties postoperatively of steroids [8], improving not only pain but also overall patient satisfaction. There are no studies, however, documenting any intra-operative effects from the use of steroids. The aim of the present study was to looking at the potential effect of pre-treatment with a Coxib on the intra-operative sevoflurane requirement. The mean end-tidal sevoflurane concentration was statistical significant different between the groups studied. No other intra or postoperative effects were, however noticed. Intra operative vital signs did not show any major differences and the decreased intra-operative anaesthesia requirement did no translate into any other difference; no significant impact on emergence time or recovery characteristics was noticed. Slightly more patients needed rescue analgesia before discharge in the post-administered group of patients. It should be acknowledge that the number of patients in the study were limited and based on a power analysis to show intra-operative effects only. Provision of oral analgesic shortly after end of anaesthesia may have a slow onset of action due to delayed enteric absorption contributing to the differences seen in postoperative rescue analgesia requirements. The results with regard to postoperative pain should also take into account that all patients had etoricoxib either before or right after surgery but also alfentanil, betamethasone at induction, local anaesthesia (bupivacaine) at wound closure and a loading dose of 30 mg/kg paracetamol orally at arrival in the phase II recovery area.

The hypothesis of the present study was that adding a Coxib, etoricoxib, prior to start of surgery would exhibit not only beneficial effects on the postoperative pain course but also exhibit intra-operative effects thereby reducing the need for main anaesthetic. Similar to what we previously found for local anaesthesia as an ankle block for Hallux Valgus surgery [4]. Intra-operative effects of NSAIDs have not been extensively studied. Ding et al have made two studies comparing ketorlolac to fentanyl during laparoscopic surgery in general anaesthesia. In the first study 60 mg ketorolac produced the same intraopeartive course as fentanyl 50 – 100 microgram [9]. In the second, they found ketorolac to be inferior to fentanyl in blunting response to incision, no major difference between the fentanyl and ketorolac group was seen in haemodynamic variables [10]. Ramirez-Ruiz et al compared ketorolac with fentanyl for MAC-sedation and in that setting ketorolac was found less effective as compared to fentanyl [11]. In another MAC-sedation study, by Yang et al, ketorolac had a significant fentanyl sparing effect [12]. Turan et al have also studied the intra-operative effects of Coxibs [13]. They found preoperative rofecoxib to have clear intra-operative effects during ENT-surgery in MAC-sedation.

There are limitations with the present study. The study design is not double-blinded; still the aim was to achieve equivalent depth of anaesthesia in both groups of patients by the use of the Cerebral State Index as an objective measure of anaesthetic depth. The cortical retrieved processed EEG index describes the balance between stimulation and anaesthetic depth [14] and also the interaction between analgesia and anaesthetics [7]. One may of course argue whether the groups were comparable with regard to depth of anaesthesia. Depth of anaesthesia is not easily defined. The introduction of EEG, depth-of-anaesthesia monitors, has made major change to anaesthetic practice. These devices make it possible to quantify the anaesthetic state in real-time on-line. Anaesthetic depth-monitors have been shown to improve anaesthetic delivery [15]. We used the Cerebral State Index to quantify depth of anaesthesia. The CSI has been shown to be more or less identical to the Bi-spectral index in determining anaesthetic depth [16,17]. We tried to make the groups as comparable as possible. We provided induction and analgesics in as standardised doses as possible, however titrating sevoflurane to maintain the EEG derived depth of anaesthesia monitoring in a rather narrow pre-defined range during surgery. A study design quit different from that of Hirota et al, where a dose of NSAIDs was added to a steady-state intra-venous anaesthesia without and surgical stimulation [18]. In disagreement with their hypothesis they could see no change in BIS from the addition of a conventional non-selective NSAID. It is, however, well known that BIS as well as CSI are both insensitive to the effects of certain anaesthetics [19,20].

It is from the present clinical study not possible to make any comments as to the potential mode of action; in what way etoricoxib interacts intra-operatively. Further studies are indeed needed in order to verify our results found in minor day surgery and to evaluate whether the intra-operative effects noticed in the present study could translate into clinical significant benefits, e.g. decrease time for recovery and decreased incidence and severity of side effects related to depth of anaesthesia such as postoperative nausea and vomiting, dizziness and fatigue; all factors of major importance to the ambulatory surgical patient satisfaction and turn over.

## Conclusion

There is today a huge and most reassuring clinical experience in that NSAIDs/Coxibs have profound effects in reducing pain, need for opioid rescue analgesia and improving patients satisfaction when used for postoperative pain management in day surgery. When added to a multi-modal pain management and provided already pre-operatively etoricoxib seems to exhibit intra-operative effects, potentially reducing the need for main anaesthetic.

## Competing interests

The authors declare that they have no competing interests.

## Authors' contributions

JJ has had the main responsibility for the study and manuscript preparation. All other authors have contributed equally.
